# Promoting Patient and Caregiver Engagement to Care in Cancer

**DOI:** 10.3389/fpsyg.2016.01660

**Published:** 2016-10-25

**Authors:** Emanuela Saita, Chiara Acquati, Sara Molgora

**Affiliations:** ^1^Department of Psychology, Università Cattolica del Sacro CuoreMilan, Italy; ^2^Graduate College of Social Work, University of HoustonHouston, TX, USA

**Keywords:** engagement, cancer, patient, caregiver, group-based intervention

## Abstract

The positive outcomes associated with Patient Engagement (PE) have been strongly supported by the recent literature. However, this concept has been marginally addressed in the context of cancer. Limited attention has also received the role of informal caregivers in promoting physical and psychological well-being of patients, as well as the interdependence of dyads. The Cancer Dyads Group Intervention (CDGI) is a couple-based psychosocial intervention developed to promote engagement in management behaviors, positive health outcomes, and the quality of the relationship between cancer patients and their informal caregivers. The article examines the ability of the CDGI to promote adaptive coping behaviors and the perceived level of closeness by comparing cancer patients participating in the intervention and patients receiving psychosocial care at usual. Results indicate that individuals diagnosed with cancer attending the CDGI present significant increases in Fighting Spirit and Avoidance, while reporting also reduced levels of Fatalism and Anxious Preoccupation. Initial indications suggest that the intervention may contribute to strengthening the relationship with the primary support person.

## Introduction

Patient engagement (PE) is defined as the cognitive, emotional, and behavioral activation of patients in their care (Graffigna et al., 2013). With increasing demands for a more active role of individuals in their healthcare (Crawford et al., 2002; Davis et al., 2005; Bellardita et al., 2012; Barello et al., 2014a,b; Menichetti et al., 2014; Barello and Graffigna, 2015), this concept is emerging as a key factor to promote healthy behaviors, better outcomes in the context of chronic diseases, as well as greater satisfaction with quality of care (Barello et al., 2012; Graffigna et al., 2013). In contrast with the great attention received elsewhere, PE has been marginally addressed within the context of cancer despite the evidence collected. For example, a recent survey conducted by CancerCare (2016) on 3000 adults highlighted that physical, emotional, financial, and social costs of cancer are not currently met because of the challenges patients report in collecting information about the disease, understanding their diagnosis, and communicating with the healthcare team. Furthermore, while PE has been traditionally promoted by focusing on individual factors, there is increasing attention to the family system (Carman et al., 2013; Donato and Bertoni, 2016). As stated by Carman et al. (2013) “those who engage and are engaged include patients, families, caregivers, and other consumers and citizens” (Carman et al., 2013, p. 224). This view of engagement as inclusive of the patient's tapestry of relationships has essential implications for cancer, since it is now well-established in the literature that cancer is a relational illness (Revenson et al., 2005; Kayser et al., 2007; Manne and Badr, 2008).

Not only the disease can negatively affect the patient's quality of life by reducing physical and psychological well-being (Epplein et al., 2011; Drake, 2012; Poghosyan et al., 2013), but the illness equally affects partners and family members, who often assume the role of informal caregivers (Meeker et al., 2011; Kim et al., 2015). While significant attention has been dedicated to cancer patients' adaptation to diagnosis and treatment, the role of caregivers has been only recently addressed by the literature (Institute of Medicine, 2008). This is in contrast with the caregiving literature, which has confirmed over the years the burden associated with this role (Given et al., 2004; Kim et al., 2006). Caregiving is often related to sleep disorders (Hearson and McClement, 2007; Fletcher et al., 2008), difficulties maintaining an occupation (Rossi Ferrario et al., 2003; Stetz and Brown, 2004; Bishop et al., 2007), emotional distress (Hagedoorn et al., 2008; Kim and Given, 2008; Kim et al., 2010), as well as high levels of anxiety and depression (Rhee et al., 2008; Cipolletta et al., 2013).

Similarly, the individual focus registered in the PE literature is antithetic to the literature on couple relationships. The extensive body of knowledge collected in the last 20 years about stress and coping has highlighted that patients' and partners' adjustment to cancer is indeed interdependent, therefore supporting the need to assume a relational perspective when working with dyads (Li and Loke, 2008; Traa et al., 2014; Vellone et al., 2014). Some authors, in fact, have identified how the couple relationship has a crucial role in promoting partners' well-being, healthy behaviors, and this datum has been confirmed across illnesses and even among healthy couples (Revenson et al., 2005; Kayser et al., 2007; Manne and Badr, 2008; Saita and Cigoli, 2009; Bertoni and Bodenmann, 2010; Bertoni et al., 2012; Carpenter et al., 2015; Donato et al., 2015; Pagani et al., 2015). The concept of dyadic coping (Bodenmann, 2005) is of particular relevance in the process to move from an individualistic to a relational view of PE. Since PE represents a dynamic and changing process, the relational context can significantly affect the individual's ability to adjust to the disease (Barello et al., 2012). It therefore follows that to support this process, the attention of the researcher should be on strengthening the coping abilities of the patient and the informal caregiver working with the dyad as a unit.

Donato and Bertoni (2016) have recently proposed a model of individual, interactive, and dyadic engagement organized on two axes: appraisal and actions of care. The authors see patient-partner healthcare patterns as a result of individual vs. shared appraisal of health management, and of an individualistic vs. relational view of the health management strategies. But how is it possible to translate this relational framework in interventions that promote patients and partners' engagement? Surprisingly, a paucity of studies have examined the association of partners' relational processes, exchanges, and engagement. Even less couple-based interventions have been recorded in the literature (Scott and Kayser, 2009; Baik and Adams, 2011; Regan et al., 2012; Badr and Krebs, 2013; see for example Badr et al., 2013). An aspect of limitation is that these experiences have focused mainly on breast, prostate, and gynecological cancers, and that only recently the literature has started to focus on other types of cancer (i.e., lung cancer). Furthermore, despite their relational focus, most contributions concentrated on patients and caregivers' outcomes separately. Hence, a significant gap in the current literature is the limited knowledge available about best practices to promote patients as well as informal caregivers' activation and engagement (Donato and Bertoni, 2016). This evidence supports the need to develop psychosocial interventions dedicated to the patient-caregiver dyad, grounded in a theoretical model that values the role of close relationships and that can be ultimately aimed at increasing the level of patients and caregivers' engagement in behaviors that (a) contribute to more beneficial adaptation to the cancer experience, and (b) support the bond between the individual and the informal caregiver.

The present contribution examines the effectiveness^1^ of the Cancer Dyads Group Intervention (CDGI); an innovative protocol developed to promote engagement in management behaviors, maximize positive health outcomes, and the quality of the relationship between cancer patients and their informal caregivers. The unique features of this approach can be identified in its theoretical framework, relational focus, and in the fact that the program can be easily translated into routine care in a variety of settings (from hospitals to community-based centers, and private practice). An overview of the program, its theoretical foundation, and techniques is available in Table 1. While participating in the CDGI program, patients, informal caregivers, and healthcare providers engage in an active partnership aimed at ensuring the best quality of care. The article presents the available empirical evidence about the ability of the CDGI to promote adaptive coping behaviors and the quality of the relationship by comparing participants and individuals receiving usual psychosocial care.

**Table 1:** **An overview of the Cancer Dyads Group Intervention**.

**The Cancer Dyads Group Intervention**
The Cancer Dyads Group Intervention (CDGI) is a supportive group-based intervention for cancer patient and caregiver dyads theoretically inspired by the Bio-psychosocial Model (Engel, 1977), the Symbolic Relational Model (Cigoli and Scabini, 2006; Scabini and Cigoli, 2012), and the Psycho-Educational Approach (Fawzy and Fawzy, 1998). The Bio-Psychosocial Model offered a holistic alternative to the biomedical model, therefore stating that the illness must be addressed focusing on three dimensions: the biological, the psychological, and the social domain (Engel, 1977). This perspective allows to contextualize care not only as the limited application of scientific knowledge (Saba, 2002), but as an action that occurs in the interaction between individuals where trust is essential (Saita et al., 2015b). As a consequence, it is possible to treat the illness while also validating the life experience of the single individual. In this sense, the ability of the individual to cope with cancer is influenced not only by the suffering of the body, but also the ability to sustain the emotional dimension of sorrow, loss, uncertainty, and –sometimes- helplessness (Saita, 2009).
The Symbolic Relational Model is the second theoretical foundation of the intervention. It is aimed at investigating family relations by focusing on the connection existing between individuals and family members (Cigoli and Scabini, 2000). Although the essence of health is perceived to be associated with the quality of the close relationships (Cigoli, 2002), only a limited number of studies have focused on the relational network. For this reason, the intervention enhances the caregiving relationship rather than the patient and the caregiver as individuals. While the first two theoretical frameworks have inspired the authors' attention to multiple determinants of health, and the crucial role the relationship with a significant other has during the time of illness, the Psycho-Educational Approach (Fawzy and Fawzy, 1998) assumes a significant value when considering patients and partner's engagement. Since it focuses on the relevance clear communication—of symptoms, treatment, and the implications on lifestyle—has in the context of illness, this aspect becomes essential when designing programs aimed at promoting patients' engagement. Moreover, the group setting has been proved to promote the emotional disclosure of participants and to facilitate the exchange and communication among its members, thus facilitating the engagement of both patients and caregivers (Saita et al., 2016).
Furthermore, the decision to use the group as a clinical tool is supported by the psychoanalytic concept of “group thought,” which refers to the experience of thinking together (Neri, 1995, 2003). Although when referring to group-based interventions it is necessary to consider numerous issues (for example: the type of group used, the kind of intervention to be planned, the techniques to be used, the setting, the strategy to conduct the sessions, and the socio-affective dynamics), it is not our intention to address here the complexity of these dimensions. The group setting becomes relevant in the development of the CDGI because the group is a psychological entity different from the sum of single individuals (Bion, 1961; Foulkes, 1964, 1973). According to Foulkes (1973), the individual unconscious is connected with the group unconscious, which the author compares to a network where each individual is metaphorically denoted by a knot. This reflection informed our idea of “thinking about oneself and the other,” which represents the basis of an intervention aimed at supporting crucial relationships during cancer, including the affective bonds the patient develops with relatives, friends, and healthcare providers.
The influence of these theoretical models has shaped and informed the techniques used in the meetings with the participants. The CDGI is organized in eight sessions and the group meets every 2 to 3 weeks for a couple of hours in a conference room of the hospital where patients are treated and where they have been recruited. Every session deals with a specific topic and begins with an exercise aimed at identifying and strengthening new coping repertoires of the dyad. The product of each exercise is later shared with the rest of the group to promote patients' and caregivers' closeness, and a sense of belonging among the participants. Two practitioners with extensive knowledge and experience in psychosocial oncology are the facilitators of the program. More practically, in collaboration with the multidisciplinary team individuals receiving care at the participating institutions are invited to participate. The group usually begins when enough dyads are recruited, from a minimum of 6 to a maximum of 10 participants. A brief overview of the CDGI is presented in the next paragraphs, while a more detailed presentation is available in a previous work (Saita et al., 2014). While the CDGI was initially developed for patients diagnosed with breast cancer, over the years the intervention has been easily adapted to meet the needs of individuals diagnosed with other types of cancer (like rare tumors; e.g., epithelial tumors of some organs or different types of sarcoma) and their caregivers. Furthermore, the CDGI has been applied not only with partners in a committed relationship, but also with other types of dyads where the role of caregiver is assumed by a member of the family system (brother, sister, daughter, or son), or a peer (for example when friends are involved).
*Session 1_My coping, your coping, our coping*
The first session is aimed at facilitating the identification of the individual's coping strategies and to develop bonds among the members of the group, and with the two conductors. After the participants are introduced to each other, the facilitators present and read the stories of two cancer patients presenting opposite coping styles: active versus avoidance and denial. By comparing their own experiences with the two proposed stories, participants are encouraged to explore the concept of coping with cancer and to recognize their own coping strategy. This is also the moment when the facilitators introduce the idea that the coping process involves the partner or significant others; a strategy to bring the concept of dyadic coping in the setting of the intervention (Acitelli and Badr, 2005; Bodenmann, 2005; Donato and Bertoni, 2016).
*Session 2_Understanding Cancer*
The session is focused on enhancing patients and caregivers' understanding of the illness. By involving a physician, it is possible for the participants to increase their knowledge about the diagnosis, treatment consequences, and overall impact on the quality of life. This is a crucial moment not only to clarify what are resources available to the patient, but also because the presence of the physician offers the opportunity to engage in an open communication which promotes the patient-provider relationship and their interaction becomes more meaningful and authentic. This meeting is divided in three main phases. In the first part, patients and caregivers can express their concerns about cancer, its treatment, and the overall cancer care continuum. The second phase involves the presence of the oncologist, who is invited to join the group to answer questions prepared by the participants or issues emerged in the first part of the meeting. This moment is particularly important to reduce the stress and uncertainty associated with cancer; especially for patients whose diagnosis is less common in the literature. Finally, dyads are invited to reflect together on the illness, to share thoughts, emotions, and concerns connected with the management of the disease. In particular, attention is given to concerns and challenges as well as to their hopes for the future.
*Session 3_Before/After*
Cancer requires the patient and the caregiver to assume new roles within the family and the relational system, with significant adjustments of the dynamics of giving and receiving care. Hence, the third session focuses on the change introduced by the diagnosis (participants are usually at the beginning the active treatment phase when they attend the intervention). Each dyad is invited to identify differences between the time before and after cancer, and later these topics are shared with the group. The clinical work of the two conductors is aimed at supporting the verbalization of concerns and aspects of change connected not only to the management of the illness, but more importantly to their relationship and the link with the supportive network (family members, close friends, colleagues); aspects which are often very difficult to verbalize and to process. As a consequence, therapists are attentive to feelings of uncertainty, resentment, denial, and inability to manage the demands of the illness. By offering participants a safe space to allow these feelings and concerns to emerge and to be shared with others facing the same stressor, it follows that participants become more aware of the impact of the illness on the life experiences of the patients, but also on the lives of caregivers, partners and family members.
*Session 4_Looking for strength and resilience through the generations*
Continuing the work to highlight the dyad's ability to engage in behaviors that facilitate a more beneficial adaptation to the cancer, as well as the relevance each other has for their well-being, the fourth session use the genogram (McGoldrick et al., 1999) as a strategy to identify strength and resilience through the generations. Participants are also asked to include relationships with significant others that may not be included in the traditional family structure. When the genogram is completed, dyads are invited to present their products to the group and to describe their family history, significant events happened to family members, and/or family myths. Finally participants are asked how the illness has been or can be integrated in the broader and larger family history.
*Session 5_ “Place me like a seal over your heart, like a seal on your arm”: the Coat of Arms*
The fifth session deepens participants' understanding of how relationships can become resources during the cancer experience by using the instrument of a family coat of arms. After providing some example, every dyad is asked to draw a coat of arms that would represent their family and its key features (some participants have even added a motto that summarized their strength and resources). The goal of the exercise is to discover positive aspects, resources and competencies already available within their close relationships, so that no resource is lost during this time of need.
*Session 6_Body Image and Cancer*
The core element of this meeting is the body and its transformation as a consequence of the illness, offering both patients and caregivers the opportunity to reflect about the beauty and strengths still present despite the negative impact of the treatment and its side effect on the body image of the patient. Using a photo-elicitation technique, each patient is invited to choose one image (from a set of 20) representing famous statues of female or male bodies (for examples the Donatello's David or the Venus de Milo), then each dyad is invited to write about the emotions associated to the image and to explore the meanings for his/her life experience. These products, which are then shared with the other participants, contribute to the discussion about body image and to the impact of cancer on intimate relationship and intimacy.
*Session 7_Mind/Body Connection*
Session seven focuses on the concept of mindfulness. It begins with a brief relaxation exercise which can be completed without any specific support (a chair is enough). Subjects are given instructions to repeat the exercise outside the setting of the intervention. The relaxation exercise introduces a reflection about the mind-body connection and the reciprocal influence, aimed at identifying strategies to handle negative emotions and the stress experienced as the end of the treatment nears. This session ends with the request to each dyad to select or create an object that symbolizes what experienced during the program and to bring it to the last session. The facilitators do the same, by selecting an object that denotes their experience as well.
*Session 8_Making Meaning and Closure*
The last session begins with the presentation of the objects the dyads have chosen or created, to support the dyad making meaning of the experience while also bringing closure to the intervention. Then, each participant is given the opportunity to verbalize what the group and the contents of the sessions may have done for him/her. Symbolically, the session ends with diplomas presented to every dyad and with a gift from the facilitators.

## Materials and methods

### Participants

Participants were 50 cancer patients recruited from two hospitals in the Northern part of Italy. Sixteen patients participated in the CDCI, while the remaining 34 were used as a control group. Cancer patients who did not participate in the group intervention were referred to usual psychosocial care available at the institutions involved in the study (psychologists and psychiatrists), where the most common type of psychosocial support is individual therapy. Institutional Review Board approval was obtained from the University IRB as coordinating center of the study and each participating institution (E. Bassini Hospital, Milano, Policlinico Hospital, Monza). The inclusion criteria for the study required that the participants were: (1) 18 years old or older, (2) free of dementia symptoms and a psychiatric diagnosis, (3), involved in a relationship with a significant other (partner, spouse, family member, friend), (4) Italian-speaking, and (5) had received a diagnosis of cancer in the last 3 months.

The average age of the participants in both groups was 62 years (*SD* = 8.80 for CDGI, *SD* = 8.12 for the Control Group). In the CDGI group, the majority of the patients were women (87.5%) diagnosed mostly with breast cancer (68.8%), where only a small number of participants were in treatment for rare cancer (31.3%). Overall, participants in the intervention group were married (56.3%) and were not highly educated (62.6% did not graduate from high school). Subjects in the control group present similar socio-demographic characteristics. Most patients were women (79.4%), and individuals with rare cancer diagnoses represented one third of the group (32.4%). Similar to what reported for the intervention group, 76.5% of cancer patients were married. However, members of the control group were more highly educated, with 32.4% being high school graduates and 8.8% being college graduates. Informal caregivers of individuals in the CDGI group were mostly romantic partners (75%), with a mean age of 65 (mean 64.8, *SD* = 9.1), low level of education (52% had only completed junior high school), and currently retired (60%).

### Procedure

Participants were initially screened by a psychologist to determine their eligibility. After a brief interview about the cancer experience, study participants completed a set of questionnaires measuring closeness with their informal caregiver, and coping strategies at time of recruitment (within 3 months from diagnosis). The same questionnaires were then completed within the 1 month after the end of the intervention, while for individuals in the control group the post-test data collection occurred 6 months after the initial contact.

### Measures

*Individual Coping*. To identify the prevailing coping style used to cope with cancer, the Italian version of the Mini-Mental Adjustment to Cancer Scale (Mini-MAC) (Watson et al., 1994; Grassi et al., 2005) was selected. The instrument is a 29-item questionnaire which identifies five coping strategies: Fighting Spirit, Hopeless/Helplessness, Anxious Preoccupation, Fatalism and Avoidance. Hopelessness/Helplessness indicates a coping style characterized by the belief of low control on events, which is associated with high levels of anxiety and depression. Individuals with a fatalistic coping behavior show low sense of control, resignation and passive acceptance of fate. Anxious Preoccupation is used to describe a coping modality with high levels of anxiety and worry about the cancer diagnosis, which can impact the quality of life of the individual. The patient is either looking for constant reassurance or is distancing herself/himself from the healthcare environment. Avoidance indicates the tendency to minimize cancer and to refrain from the search of information. Fighting Spirit is characterized by an optimistic attitude toward one's ability to cope with the illness. Next to low levels of anxiety and depression, individuals presenting fighting spirit tend to perceive the illness as a challenge. They implement diverse and flexible cognitive strategies, which contribute to a positive appraisal of the experience. This coping style has been associated with better psychological morbidity, increased sense of control and better prognosis (Pettingale et al., 1985; Burgess et al., 1988; Saita et al., 2015a).

*Interpersonal Closeness*. Perceived level of closeness with the primary support person was measured by the Inclusion of the Other in the Self (IOS) Scale (Aron et al., 1992). The measure consists of seven pairs of overlapping circles that are drawn to show varying levels of overlap, indicating an increasing degree of closeness in the relationship. A 7-point scale is used to score the degree of closeness. In the present study, we asked the individual to indicate up to five persons who provide support to them and describe each relationship by choosing one of the seven circles. Although the scale is not formally validated in the Italian population, the very easy and intuitive nature of the questionnaire -being a single item pictorial tool- has contributed to its use with Italian subjects, as documented by earlier works of these authors (Saita et al., 2015a), other Italian researchers (ex. De Panfilis et al., 2015), or studies conducted including Italian samples (Karremans et al., 2011).

### Data analysis

Descriptive statistics of cancer patients were obtained for all the variables compiling frequency tables, histograms, and bar graphs. Differences in the coping style behavior for the intervention and control group were examined with Independent Sample *t*-test, while differences within patients were assessed calculating paired samples *t*-test. IBM SPSS Statistics 22 was used for data screening and data analysis. Changes in the perceived degree of closeness with the informal caregivers were described comparing who were the sources of support identified by the cancer patients and by calculating the mean scores originating from the position of the IOS Scale used to describe these relationships

## Results

### Differences between patients: examining changes in coping style between the intervention and control group at pre-test and post-test

An Independent Samples *t*-test was used to compare the mean score of each coping strategy of individuals participating in the CDGI intervention and those of the control group at pre and post-test, in order to identify if the two groups were already different in their coping behaviors at pre-test and if a change occurred at the post-test. While results indicate that no statistically significant difference existed at pre-test, at post-test individuals who participated in the CDGI presented significantly higher Fighting Spirit [*t*_(48)_ = 2.71, *p* < 0.01] than cancer patients who received usual psychosocial care.

### Differences within patients: pre-test/post-test comparison among the participants of the intervention and control group

The previous findings were confirmed when a pre-test/post-test comparison was conducted on each group, using Paired Samples *t*-test. Results indicate that individuals diagnosed with cancer attending the CDGI present significant increase in Fighting Spirit [*t*_(14)_ = −2.31, *p* < 0.05] and Avoidance [*t*_(14)_ = −4.65, *p* < 0.001], while reporting also reduced mean scores in Fatalism [*t*_(14)_ = 3.42, *p* < 0.01] and Anxious Preoccupation [*t*_(14)_ = 3.40, *p* < 0.01]. On the contrary, the changes registered in the control group indicate that individuals reported significantly higher scores of Hopelessness/Helplessness [*t*_(32)_ = −2.41, *p* < 0.05] next to reduced Fatalism [*t*_(32)_ = 4.54, *p* < 0.001] (Tables 2, 3).

**Table 2:** **CDGI Pre-post test comparison**.

						**95% CI**	
**Variable**	**Time**	**Mean**	***SD***	***t***	***p***	**LL**	**UL**
Fighting spirit	Pre-test	2.99	0.79592	−2.308	**0.036**	−0.84150	−0.03350
	Post-test	3.43	0.50091				
Hopelessness/Helplessness	Pre-test	1.67	0.55812	−2.844	**0.012**	−1.40003	−0.20059
	Post-test	2.47	0.79120				
Fatalism	Pre-test	2.96	0.64174	3.424	**0.004**	0.411759	1.769491
	Post-test	1.87	0.818325				
Anxious preoccupation	Pre-test	2.14	0.52599	3.402	**0.004**	0.077864	0.339011
	Post-test	1.93	0.648717				
Avoidance	Pre-test	2.39	0.59139	−4.652	**0.000**	−0.934138	−0.347112
	Post-test	3.03	0.442672				

*Bold values indicates significant results*.

**Table 3:** **Control Group Pre-post test comparison**.

						**95% CI**	
**Variable**	**Time**	**Mean**	***SD***	***t***	***p***	**LL**	**UL**
Fighting spirit	Pre-test	2.77	0.63038	−1.327	0.194	−0.45831	0.09654
	Post-test	2.95	0.61335				
Hopelessness/Helplessness	Pre-test	1.86	0.60824	−2.410	**0.022**	−0.96772	−0.08169
	Post-test	2.38	1.01721				
Fatalism	Pre-test	2.70	0.69186	4.537	**0.000**	0.467034	1.226496
	Post-test	1.86	0.735365				
Anxious preoccupation	Pre-test	2.09	0.70088	0.880	0.385	−0.153928	0.388772
	Post-test	1.97	0.556457				
Avoidance	Pre-test	2.67	0.87083	−0.352	0.727	−0.478033	0.337150
	Post-test	2.74	0.715798				

*Bold values indicates significant results*.

While the results about Hopelessness/Helplessness may be considered in contrast with the overall aim of the intervention, they may be contextualized referring to the types of cancer included in the study. Given the limited number of subjects in the two groups, this analysis is only exploratory in nature. When the mean scores have been compared differentiating between breast and rare cancer patients, results indicate that at pre-test individuals with rare tumors in the CDGI presented significantly higher scores of Hopelessness/Helplessness [*t*_(14)_ = −2.71, *p* < 0.05] compared to women with breast cancer, while on the contrary this group scored higher on Fighting Sprit [*t*_(14)_ = 2.63, *p* < 0.05] and Fatalism [*t*_(14)_ = 2.88, *p* < 0.05]. The same differences were found also in the control group, where breast cancer patients also shown higher Avoidance [*t*_(32)_ = 2.75, *p* < 0.05]. At post-test, it clearly emerges how the intervention contributed to reduced Hopelessness in patients with rare tumors [*t*_(14)_ = 4.19, *p* < 0.01], while patients with breast cancer presented significantly higher Fighting Spirit [*t*_(14)_ = 4.15, *p* < 0.01] and significantly lower Fatalism [*t*_(14)_ = −3.9, *p* < 0.01]. In the control group, differences were registered on Fatalism [*t*_(32)_ = −6.2, *p* < 0.001] and Hopelessness [*t*_(32)_ = 7.7, *p* < 0.001], with individuals with breast cancer presenting higher Hopelessness and lower Fatalism (Tables 4, 5).

**Table 4:** **Pre-test comparison by cancer type**.

							**95% CI**	
**Group**	**Variable**	**Cancer Type**	**Mean**	***SD***	***t***	***p***	**LL**	**UL**
CDGI	Fighting spirit	Breast cancer	3.29	0.70550	**2.63**	**0.02**	0.17549	1.73542
		Rare Tumor	2.34	0.58885			0.19381	1.71710
	Hopelessness/Helplessness	Breast cancer	1.46	0.40268	**−2.71**	**0.02**	−1.22514	−0.14268
		Rare tumor	2.15	0.60063			−1.41430	0.04648
	Fatalism	Breast cancer	3.21	0.59635	**2.88**	**0.01**	0.20947	1.42689
		Rare tumor	2.40	0.28284			0.34622	1.29014
	Anxious preoccupation	Breast cancer	2.12	0.61938	−0.25	0.74	−0.70127	0.55563
		Rare tumor	2.19	0.27181			−0.55076	0.40513
	Avoidance	Breast cancer	2.43	0.52549	0.40	0.69	−0.57228	0.83591
		Rare tumor	2.30	0.77862			−0.81509	1.07873
Control Group	Fighting spirit	Breast cancer	3.03	0.58535	**5.46**	**<0.001**	0.42533	1.18534
		Rare tumor	2.22	0.27372			0.50522	1.10545
	Hopelessness/Helplessness	Breast cancer	1.67	0.53597	**−2.88**	**0.007**	−0.98894	−0.16545
		Rare tumor	2.25	0.58392			−1.01474	−0.13964
	Fatalism	Breast cancer	2.91	0.68444	**2.75**	**0.010**	0.16479	1.10857
		Rare tumor	2.27	0.49736			0.21151	1.06185
	Anxious preoccupation	Breast cancer	2.03	0.79625	−0.69	0.492	−0.70749	0.34753
		Rare tumor	2.21	0.44962			−0.61719	0.25724
	Avoidance	Breast cancer	2.89	0.93844	**2.75**	**0.010**	0.05553	1.28487
		Rare tumor	2.22	0.48047			0.17412	1.16628

*Bold values indicates significant results*.

**Table 5:** **Post-test comparison by cancer type**.

							**95% CI**	
**Group**	**Variable**	**Cancer Type**	**Mean**	***SD***	***t***	***p***	**LL**	**UL**
CDGI	Fighting spirit	Breast cancer	3.67	0.33344	**4.15**	**0.001**	0.37573	1.17882
		Rare tumor	2.90	0.37914			0.31064	1.24390
	Hopelessness/Helplessness	Breast cancer	2.86	0.58485	**4.20**	**0.001**	0.60317	1.86411
		Rare tumor	1.63	0.42953			0.65690	1.81037
	Fatalism	Breast cancer	1.48	0.666742	**−3.90**	**0.002**	−1.912	−0.555328
		Rare tumor	2.72	0.303315			−1.753	−0.713319
	Anxious preoccupation	Breast cancer	1.88	0.744678	−0.45	0.65	−0.934735	0.607462
		Rare tumor	2.05	0.410792			−0.789682	0.462409
	Avoidance	Breast cancer	3.09	0.314498	0.78	0.44	−0.327733	0.709551
		Rare tumor	2.90	0.675463			−0.632810	1.014628
Control Group	Fighting spirit	Breast cancer	3.02	0.68621	1.00	0.32	−0.23184	0.68401
		Rare tumor	2.80	0.40927			−0.15970	0.61187
	Hopelessness/Helplessness	Breast cancer	2.90	0.79322	**7.71**	**<0.001**	1.06471	2.10328
		Rare tumor	1.31	0.40415			1.16541	2.00258
	Fatalism	Breast cancer	1.48	0.450557	**−6.24**	**<0.001**	−1.521	−0.772675
		Rare tumor	2.63	0.598787			−1.5795	−0.714950
	Anxious preoccupation	Breast cancer	1.91	0.492031	−0.90	0.37	−0.600940	0.232482
		Rare tumor	2.09	0.681147			−0.673038	0.304580
	Avoidance	Breast cancer	2.77	0.635979	0.25	0.80	−0.476157	0.608370
		Rare tumor	2.70	0.893156			−0.573572	0.705786

*Bold values indicates significant results*.

### Examining changes in the perceived degree of closeness with the primary support person

The relational perspective of the intervention determines the need to explore the perceived level of closeness with the informal caregiver. To examine changes in the two groups we first considered which persons were identified as the primary source of support, and then we compared the mean values obtained from the picture selected by the participants as indication of closeness. For individuals in the CDGI, the source of their support is identified in the relationship with partners, children and siblings. This indication is confirmed also at post-test, with these three categories being the most listed by the participants. One patient also indicated that the family as a whole became the source of support. For the control group, while at pre-test the most commonly identified individuals were partners, children, and siblings, 6 months after the initial contact patients started to expand their supportive network and to include parental figures and the healthcare system.

**Figure 1:**
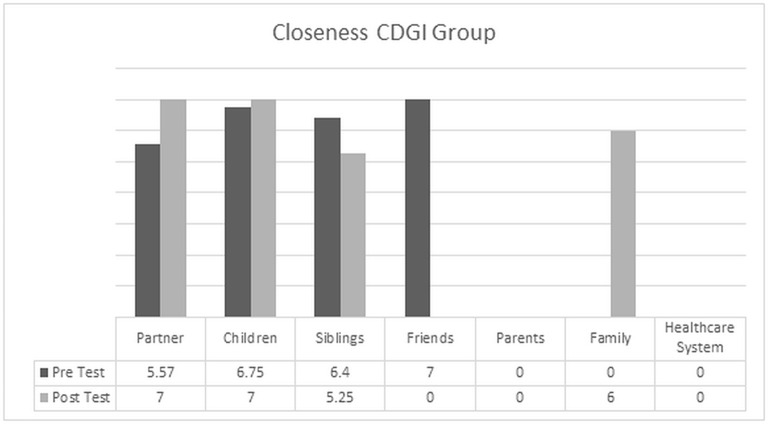
**Pretest/Posttest comparision of the mean value of closeness for individuals in the CDGI**.

**Figure 2:**
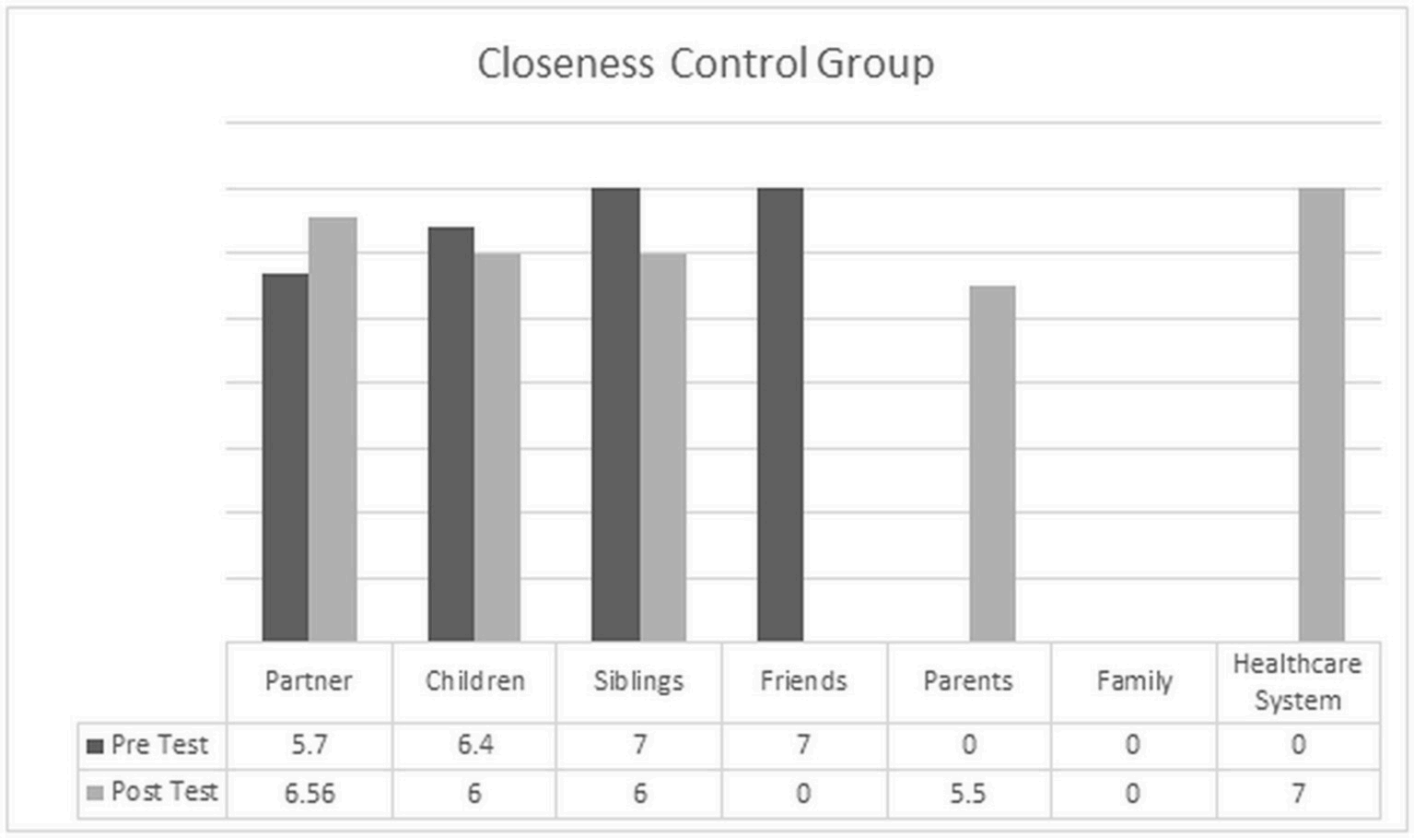
**Pretest/Posttest comparision of the mean value of closeness for the control group**.

When focusing specifically on the degree of closeness with the primary support person, which are presented in Figures 1, 2, it is possible to notice how members of the CDGI reported elevated levels of closeness with the primary support person; as indicated by higher scores in the relationships with partners, children and siblings. We want also to note that while the perceived closeness with friends was very elevated at pre-test, this relationship was no longer indicated at post-test. On the contrary, friends were substituted by the family as a whole, indicating greater reliance of the patient on the family system.

Figure 2 illustrates the change registered among individuals in the control group. The perceived level of closeness with the partner increased, but for those patients who identified the primary support person with children or siblings, the perceived closeness was reduced at post-test. Furthermore, the relationship with peers lost relevance over time, substituted by a closer connection with the parental figures. It is also important to highlight that for this group the family as a whole did not assume a meaningful role over time, and that external sources of support were searched in the relationships established with healthcare providers, such as physicians, nurses, and mental health professionals.

## Discussion

In this paper we have supported the relevance close relationships have in the promotion of PE in the context of cancer. As a result of the need to assume a relational view of this concept, we have illustrated the CDGI as a way to operationalize PE as involving patients and informal caregivers. This program has been developed with the goal to increase patients' engagement in management behaviors, enhance awareness of the emotional dimension associated with the illness, and to promote the quality of the relationship between cancer patients and their informal caregivers. Furthermore, this article aspired to provide further empirical evidence about the effectiveness of the CDGI to activate patients' coping abilities and strengthening the senses of closeness with the primary support person, while previous works have been mostly focused on the process of the intervention (Saita et al., 2014). The analysis conducted in the present contribution focused on the coping strategies and the perceived degree of closeness, by comparing pre-test and post-test scores of patients who participated in the intervention and those of were referred to usual psychosocial care. Three are the most relevant findings to discuss.

First, differences in coping strategies between patients highlight how individuals who participated in the CDGI have developed higher levels of Fighting Spirit at the end of the program, which suggests that participants were able to develop more adaptive behavioral and emotional strategies. They seem to be better equipped to cope with the potentially stressful events and feelings associated with the cancer experience than the individuals who did not. Moreover, the enactment of coping behaviors characterized by Fighting Spirit also reveals the willingness of the individual to face the multiple stressors a cancer diagnosis originates, and the realization of mastering the abilities necessary to face the disease. This aptitude also sustains the hope to foresee a future with no cancer, despite the uncertainty associated with this illness (Coward and Kahn, 2004; Saita et al., 2015b).

This finding is also supported by the pre-test and post-test comparison of the two groups. In our study, the control group reported statistically significant increase in Hopelessness and Fatalism. Individuals receiving usual care showed over time indication of low control on events, resignation, and passive acceptance, which has been associated with negative quality of life outcomes in the literature (Meerwein, 1989; Barraclough, 2001). On the contrary, individuals in the intervention group presented aspects of change in all the coping styles evaluated. While the indication of higher Fighting Spirit and Avoidance, reduced Fatalism, and lower Anxious Preoccupation are in line with the outlined goals, the statistically significant increase in Hopelessness/Helplessness appeared in contrast with our hypotheses. However, these findings may be clarified considering how the two types of cancer in the sample (breast cancer vs. rare tumors) can affect the quality of life of the individual, given the dissimilar treatments, outcomes, and survivorship issues. Although it is necessary to be mindful of the very limited number of individuals with rare tumors included in the study, it is possible that the different level of information and knowledge available about rare types of cancer (including the preparedness of the healthcare team) can contribute to affect the outcome of the intervention. A second consideration then pertains the feelings associated with a cancer diagnosis, which are often unexpected and destabilizing for the individual, whose sense of psychological and physical integrity is suddenly threatened. Despite the differences between facing a well-known and studied pathology vs. a rare disease, the possibility to receive information related to the illness and its treatment contributes to higher adherence (Cousson-Gélie et al., 2008). In this sense, the higher level of Hopelessness/Helplessness registered among patients in the control group can be partially explained by the sense of loneliness and isolation experienced when facing cancer alone. The attendance of the CDGI can, on the contrary, alleviate the feelings of helplessness and lack of control the diagnosis has originated.

Finally, given the theoretical foundation of our work and the role close relationships have for physical and psychological well-being of patients and informal caregivers, we examined patients' closeness. These findings suggest that the CDGI experience represents a setting where the relationship with the primary support person can be nurtured and strengthened. Not only CDGI participants continued to identify as sources of support the relationships established within the family of origin or the partner, but the descriptive analysis about the mean level of closeness indicates that the program contributed to increased degree of proximity and support with the informal caregiver and the family as a whole. Differently, individuals in the control group experienced lower sense of closeness with children and siblings, and some of them showed a tendency over time to rely more on the healthcare system. This movement mirrors “the stress-coping cascade effect” (Bodenmann, 2005), which describes how individual coping strategies are substituted by dyadic approaches (involving partners, relatives, and friends), and ultimately healthcare professionals. Hence, when a coping strategy is no longer functional, the individual continues to search support until he can find an adequate response to his needs. Results of the descriptive analysis of the IOS Scale data are of particular interest because while extensive attention has been given to the development of psychosocial interventions to promote coping, a limited number of studies have investigated the less conscious aspect of the relationship between cancer patient and his/her informal caregiver (Aron and Aron, 1986; Aron et al., 1991).

Given the important clinical implications the intervention has for the current debate about best-practices to promote patients and informal caregivers' engagement to care, it is important to describe how this contribution is affected by several limitations. First, the present work relied on a small sample size, which limits our ability to generalize these findings and also represents a limitation in the selection of the data analysis strategy. While difficulties in the recruitment of dyads in research are extensively reported, this article also represents the result of years of collaboration with hospital settings and illustrates a strategy to move from a qualitative analysis of the intervention (Saita et al., 2014) toward a quantitative approach. Moreover, in this contribution it was not possible to include and analyze data from both members of the dyad. Although this may seem in contrast with the relational perspective that has inspired our work, the need to focus only on patients' data was influenced by the fact that the questionnaires completed by the participating caregivers, although being collected as part of the research protocol, were not fully available at the time of the analysis. It will be therefore important to include data about patients and caregivers' change as part of the participation in the CDGI to provide further empirical evidence to the findings we have published so far. Third, as the intervention promotes dyadic coping strategies to strengthen adaptation and engagement in both partners, a measure of dyadic coping should be included in future works. Similarly, it will be critical to add a measure of PE and to target participants' satisfaction with care; aspects that were not investigated in the current work. Finally, from a methodological perspective, data should be analyzed using a dyadic data approach, in order to account for the interdependence of patients' and partners' scores.

Summarizing, while a relational view of the concept of PE has received increasing attention in the literature, the present work has illustrated how it is possible to develop interventions that support the bond between the individual and his/her informal caregiver. Within these experiences, the CDGI contributes to foster PE as a “process-like and multidimensional experience” (Graffigna et al., 2014, p. 1), by focusing both on health information (working together with providers) and on the affective dimension of the illness. Participants reported significant reduction in coping strategies like Anxious Preoccupation and Fatalism, and higher Fighting Spirit than controls. Furthermore, from this initial analysis it is possible to appreciate how the CDGI represents a setting where it is possible to support the relationship between patient and informal caregiver.

Not only this program is almost unique in the field of PE in the context of cancer, but while previous studies have targeted only individuals outcomes, the intervention described here helps participants to find the resources to cope with the illness indentifying the relationship as the therapeutic tool. Since support within the context of a close relationship leads to better outcomes, we propose that forms of intervention that focus on these dyads would be appropriate and potentially effective in promoting and enhancing engagement and quality of life for both the patient and partner (Kayser and Scott, 2008; Saita et al., 2014). This brings us back to the suggestions identified by Donato and Bertoni (2016) about the development of interventions aimed at promoting patients and partners' enagement. The authors discuss the relevance of a clear theoretical framework, of addressing both patients and partner's needs, and to being able to integrate individual, interactive, and relational levels of intervention. These indications guided our work and were used to support the effectiveness of the intervention, given that the CDGI integrates the Bio-psychosocial Model (Engel, 1977), the Symbolic Relational Model (Cigoli and Scabini, 2006; Scabini and Cigoli, 2012), and the Psycho-Educational Approach (Fawzy and Fawzy, 1998). Similarly, the intervention focuses on the dyad and the relationship between patients and caregiver, therefore integrating excercises and activities with individual, interactive and relational focus. Our study then represents a nice integration of their model, as it introduces also a third dimension to consider when developing interventions to promote PE using a relational perspective. As the appraisal of care and the actions of care define three levels of patient-partner engagement, psychosocial interventions should be placed along a continuum of settings (ranging from individual, couple, and group of patients or caregivers, and groups of dyads) with the goal to offer the most appropriate and effective setting of intervention given the unique characteristics of the participants the equipe is working with.

## Author contributions

Literature Review: CA and SM. Description of Intervention: ES. Data collection and Data analysis: ES and CA. Discussion: All authors.

### Conflict of interest statement

The authors declare that the research was conducted in the absence of any commercial or financial relationships that could be construed as a potential conflict of interest.

## Footnotes

^1^Cfr. (Haynes, 1999).

## References

[B1] Acitelli L. K. Badr H. (2005). My illness or our illness? Attending to the relationship when one partner is ill, in Couples Coping with Stress: Emerging Perspectives on Dyadic Coping, eds Revenson T. A. Kayser K. Bodenmann G. (Washington, DC: American Psychological Association), 121–136.

[B2] Aron A. Aron E. N. (1986). Love as the Expansion of Self: Understanding Attraction and Satisfaction. New York, NY: Hemisphere.

[B3] Aron A. Aron E. N. Smollan D. (1992). Inclusion of the other in the self scale and the structure of interpersonal closeness. J. Pers. Soc. Psychol. 63, 596–612. 10.1037/0022-3514.63.4.596

[B4] Aron A. Aron E. N. Tudor M. Nelson G. (1991). Close relationship as including other in the self. J. Pers. Soc. Psychol. 60, 241–253. 10.1037/0022-3514.60.2.241

[B5] Badr H. Camack C. L. Milbury K. Temech M. (2013). Psychosocial intervention for couples coping with cancer: A systematic review, in Psychological Aspects of Cancer, eds Carr B. I. Steel J. (New York, NY: Springer), 177–198.

[B6] Badr H. Krebs P. (2013). A systematic review and meta-analysis of psychosocial interventions for couples coping with cancer. Psychooncology 22, 1688–1704. 10.1002/pon.3200 23045191 PMC3562417

[B7] Baik O. M. Adams K. B. (2011). Improving the well-being of couples facing cancer: a review of couples-based psychosocial intervention. J. Marital Fam. Ther. 37, 250–266. 10.1111/j.1752-0606.2010.00217.x 21457288

[B8] Barello S. Graffigna G. (2015). Engagement-sensitive decision making: training doctors to sustain patient engagement in medical consultations, in Patient Engagement: A Consumer-Centered Model to Innovate Healthcare, eds Graffigna G. Barello S. Triberti S. (Berlin: De Gruyter Open), 78–93.

[B9] Barello S. Graffigna G. Savarese M. Bosio A. C. (2014a). Engaging patients in health management: towards a preliminary theoretical conceptualization. Psicol. Salute 3, 11–33. 10.3280/PDS2014-003002

[B10] Barello S. Graffigna G. Vegni E. (2012). Patient engagement as an emerging challenge for healthcare services: mapping the literature. Nurs. Res. Pract. 1, 17 10.1155/2012/905934 PMC3504449 23213497

[B11] Barello S. Graffigna G. Vegni E. Bosio A. C. (2014b). The challenges of conceptualizing patient engagement in health care: a lexicographic literature review. J. Particip. Med. 6:e9.

[B12] Barraclough J. (ed.). (2001). Integrated Cancer Care. Oxford: Oxford University Press.

[B13] Bellardita L. Graffigna G. Donegani S. Villani D. Villa S. Tresoldi V. (2012). Patient's choice of observational strategy for early-stage prostate cancer. Neuropsychol. Trends 12, 107–116. 10.7358/neur-2012-012-bell

[B14] Bertoni A. Bodenmann G. (2010). Satisfied and dissatisfied couples: positive and negative dimensions, conflict styles, and relationships with family of origin. Eur. Psychol. 15, 175–184. 10.1027/1016-9040/a000015

[B15] Bertoni A. Parise M. Iafrate R. (2012). Beyond satisfaction: Generativity as a new outcome of couple functioning, in Marriage: Psychological Implications, Social Expectations, and Role of Sexuality, eds Esposito P. E. Lombardi C. I. (New York, NY: Nova Science Publishers), 115–131.

[B16] Bion W. R. (1961). Experiences in Groups. London: Tavistock Publications.

[B17] Bishop M. M. Beaumont J. L. Hahn E. A. Cella D. Andrykowski M. A. Brady M. J.. (2007). Late effects of cancer and hematopoietic stem-cell transplantation on spouses or partners compared with survivors and survivor-matched controls. J. Clin. Oncol. 25, 1403–1411. 10.1200/JCO.2006.07.5705 17416860

[B18] Bodenmann G. (2005). Dyadic coping and its significance for marital functioning, in Couples Coping with Stress: Emerging Perspectives on Dyadic Coping, eds Revenson T. A. Kayser K. Bodenmann G. (Washington, DC: American Psychological Association), 33–50.

[B19] Burgess C. Morris T. Pettingale K. W. (1988). Psychological response to cancer diagnosis II. Evidence for coping styles. J. Psychosom. Res. 20, 795–802. 10.1016/0022-3999(88)90067-0 3184015

[B20] CancerCare (2016). 2016 CancerCare Patient Access and Engagement Report. Available online at http://www.cancercare.org/accessengagementreport

[B21] Carman K. L. Dardess P. Maurer M. Sofaer S. Adams K. Bechtel C.. (2013). Patient and family engagement: a framework for understanding the elements and developing interventions and policies. Health Aff. 32, 223–231. 10.1377/hlthaff.2012.1133 23381514

[B22] Carpenter D. M. Elstad E. A. Sage A. J. Geryk L. L. DeVellis R. F. Blalock S. J. (2015). The relationship between partner information-seeking, information-sharing, and patient medication adherence. Patient Educ. Couns. 98, 120–124. 10.1016/j.pec.2014.10.001 25455797 PMC4314448

[B23] Cigoli V. (2002). Lo spirito della relazione e la provocazione della malattia, in Il Medico, la Famiglia e la Comunità. L'approccio Biopsicosociale alla Salute e alla Malattia, eds Cigoli V. Mariotti M. (Milano: Franco Angeli), 56–76.

[B24] Cigoli V. Scabini E. (2000). *Il Famigliare*. Legami, Simboli e Transizioni. Milano: Raffaello Cortina.

[B25] Cigoli V. Scabini E. (2006). Family Identity. Ties, Symbols, and Transitions. New York, NY: Erlbaum.

[B26] Cipolletta S. Shams M. Tonello F. Pruneddo A. (2013). Caregivers of patients with cancer: anxiety, depression, and distribution of dependency. Psychooncology 22, 133–139. 10.1002/pon.2081 23296634

[B27] Cousson-Gélie F. Vernejoux J. M. Bazex-Chanteloube H. Raherison C. Ozier A. Girodet P. O.. (2008). Multi-professional lung cancer disclosure to change anxiety and depression: an exploratory study. Thorax 63, 658. 10.1136/thx.2007.093922 18587039

[B28] Coward D. D. Kahn D. L. (2004). Resolution of spiritual disequilibrium by women newly diagnosed with breast cancer. Oncol. Nurs. Forum 31, E24–E31. 10.1188/04.ONF.E24-E31 15017451

[B29] Crawford M. J. Rutter D. Manley C. Weaver T. Bhui K. Fulop N.. (2002). Systematic review of involving patients in the planning and development of health care. Br. Med. J. 325, 1263–1265. 10.1136/bmj.325.7375.1263 12458240 PMC136920

[B30] Davis K. Schoenbaum S. C. Audet A. M. (2005). A 2020 vision of patient-centered primary care. J. Gen. Intern. Med. 20, 953–957. 10.1111/j.1525-1497.2005.0178.x 16191145 PMC1490238

[B31] De Panfilis C. Riva P. Preti E. Cabrino C. Marchesi C. (2015). When social inclusion is not enough: implicit expectations of extreme inclusion in borderline personality disorder. Personal. Disord. 6, 301–309. 10.1037/per0000132 26147068

[B32] Donato S. Bertoni A. (2016). A relational perspective on patient engagement: Suggestions from couple-based research and intervention, in Transformative Healthcare Practice through Patient Engagement, ed Graffigna G. (IGI Global), 83–106.

[B33] Donato S. Parise M. Iafrate R. Bertoni A. Finkenauer C. Bodenmann G. (2015). Dyadic coping responses and partners' perceptions for relationship satisfaction: an actor-partner longitudinal analysis. J. Soc. Pers. Relat. 32, 580–600. 10.1177/0265407514541071

[B34] Drake K. (2012). Quality of life for cancer patients: from diagnosis to treatment and beyond. Nurs. Manage. 43, 20–25. 10.1097/01.NUMA.0000410865.48922.18 22240919

[B35] Engel G. (1977). The need for a new medical model: a challenge for biomedicine. Science 196, 129–136. 10.1126/science.847460 847460

[B36] Epplein M. Zheng Y. Zheng W. Chen Z. Gu K. Penson D.. (2011). Quality of life after breast cancer diagnosis and survival. J. Clin. Oncol. 29, 406–412. 10.1200/JCO.2010.30.6951 21172892 PMC3058286

[B37] Fawzy F. I. Fawzy N. W. (1998). Group therapy in the cancer setting. J. Psychosom. Res. 45, 191–200. 10.1016/S0022-3999(98)00015-4 9776366

[B38] Fletcher B. S. Paul S. M. Dodd M. J. Schumacher K. West C. Cooper B.. (2008). Prevalence, severity, and impact of symptoms on female family caregivers of patients at the initiation of radiation therapy for prostate cancer. J. Clin. Oncol. 26, 599–605. 10.1200/JCO.2007.12.2838 18235118

[B39] Foulkes S. H. (1964). Therapeutic Group Analysis. Reprinted 1984. London: Karnac Books.

[B40] Foulkes S. H. (1973). The group as a matrix of the individual's mental life, in Selected Papers, ed Foulkes E. (London: Karnac Books), 223–233.

[B41] Given B. Wyatt G. Given C. Gift A. Sherwood P. DeVoss D.. (2004). Burden and depression among caregivers of patients with cancer at the end of life. Oncol. Nurs. Forum 31, 1105–1117. 10.1188/04.ONF.1105-1117 15547633 PMC1315286

[B42] Graffigna G. Barello S. Libreri C. Bosio C. A. (2014). How to engage type-2 diabetic patients in their own health management: implications for clinical practice. BMC Public Health 14:648. 10.1186/1471-2458-14-648 24966036 PMC4083034

[B43] Graffigna G. Barello S. Riva G. (2013). Technologies for patient engagement. Health Aff. 32, 1172 10.1377/hlthaff.2013.0279 23733998

[B44] Grassi L. Buda P. Cavana L. Annunziata M. A. Torta R. Varetto A. (2005). Styles of coping with cancer: the Italian version of the mini-mental adjustment to cancer (Mini-MAC) scale. Psychooncology 14, 115–124. 10.1002/pon.826 15386782

[B45] Hagedoorn M. Sanderman R. Bolks H. N. Tuinstra J. Coyne J. C. (2008). Distress in couples coping with cancer: a meta-analysis and critical review of role and gender effect. Psychol. Bull. 134, 1–30. 10.1037/0033-2909.134.1.1 18193993

[B46] Haynes B. (1999). Can it work? Does it work? Is it worth it? The testing of healthcare interventions is evolving. BMJ 319, 652–653. 10.1136/bmj.319.7211.652 10480802 PMC1116525

[B47] Hearson B. McClement S. (2007). Sleep disturbance in family caregivers of patients with advanced cancer. Int. J. Palliat. Nurs. 13, 495–501. 10.12968/ijpn.2007.13.10.27493 18073709

[B48] Institute of Medicine (2008). Cancer Care for the Whole Patient: Meeting Psychosocial Health Needs. Washington, DC: The National Academies Press. 20669419

[B49] Karremans J. C. Regalia C. Paleari F. G. Fincham F. D. Cui M. Takada N. (2011). Maintaining harmony across the globe: the cross-cultural association between closeness and interpersonal forgiveness. Soc. Psychol. Pers. Sci. 2, 443–451. 10.1177/1948550610396957

[B50] Kayser K. Scott J. L. (2008). Helping Couples Cope with Women's Cancers. An Evidence-Based Approach for Practitioners. New York, NY: Springer.

[B51] Kayser K. Watson L. E. Andrade J. T. (2007). Cancer as a “we-desease”: examining the process of coping from a relational perspective. Fam. Syst. Health 25, 404–418. 10.1037/1091-7527.25.4.404

[B52] Kim Y. Baker F. Spillers R. L. Wellisch D. K. (2006). Psychological adjustment of cancer caregivers with multiple roles. Psychooncology 15, 795–804. 10.1002/pon.1013 16502472

[B53] Kim Y. Given B. A. (2008). Quality of life of family caregivers of cancer survivors across the trajectory of the illness. Cancer 112, 2556–2568. 10.1002/cncr.23449 18428199

[B54] Kim Y. Kashy D. A. Spillers R. L. Evans T. V. (2010). Needs assessment of family caregivers of cancer survivors: three cohorts comparison. Psychooncology 19, 573–582. 10.1002/pon.1597 19582798

[B55] Kim Y. Shaffer K. M. Carver C. S. Cannady R. S. (2015). Quality of life of family caregivers eight years after a relative's cancer diagnosis: follow-up of the national quality of life survey for caregivers. Psychooncology 25, 266–274. 10.1002/pon.3843 25976620

[B56] Li Q. Loke A. Y. (2008). A systematic review of spousal couple-based intervention studies for couples coping with cancer: direction for the development of interventions. Psychooncology 23, 731–739. 10.1002/pon.3535 24723336

[B57] Manne S. Badr H. (2008). Intimacy and relationship processes in couples' psychosocial adaptation to cancer. Cancer 112, 2541–2555. 10.1002/cncr.23450 18428202 PMC4449141

[B58] McGoldrick M. Gerson R. Shellenberger S. (1999). Genograms: Assessment and Intervention, 2nd Edn. New York, NY: Norton.

[B59] Meeker M. A. Finnell D. Othman A. K. (2011). Family caregivers and cancer pain management: a review. J. Fam. Nurs. 17, 29–60. 10.1177/1074840710396091 21343621

[B60] Meerwein F. (1989). Psicologia e oncologia. Torino: Bollati Borighieri.

[B61] Menichetti J. Libreri C. Lozza E. Graffigna G. (2014). Giving patients a starring role in their own care:a bibliometric analysis of the on-going literature debate. Health Expect. 19, 516–526. 10.1111/hex.12299 25369557 PMC5055237

[B62] Neri C. (1995). Pensiero di gruppo. Koinos Quad. 2, 73–85.

[B63] Neri C. (2003). Gruppo. Roma: Edizioni Borla.

[B64] Pagani A. F. Donato S. Parise M. Iafrate R. Bertoni A. Schoebi D. (2015). When good things happen: the effects of implicit and explicit capitalization attempts on intimate partners' well-being. Fam. Sci. 6, 119–128. 10.1080/19424620.2015.1082013

[B65] Pettingale K. W. Morris T. Greer S. Haybittle J. L. (1985). Mental attitudes to cancer: an addictional prognostic factor. Lancet 30, 750–762. 10.1016/S0140-6736(85)91283-8 2858012

[B66] Poghosyan H. Sheldon L. K. Leveille S. G. Cooley M. E. (2013). Health-related quality of life after surgical treatment in patients with non-small cell lung cancer: a systematic review. Lung Cancer 81, 11–26. 10.1016/j.lungcan.2013.03.013 23562675

[B67] Regan T. W. Lambert S. D. Girgis A. Kelly B. Kayser K. Turner J. (2012). Do couple-based interventions make a difference for couples affected by cancer? A systematic review. BMC Cancer 12:279. 10.1186/1471-2407-12-279 22769228 PMC3464780

[B68] Revenson T. A. Kayser K. Bodenmann G. (eds.) (2005). Couples Coping with Stress: Emerging Perspectives on Dyadic Coping. Washington, DC: American Psychological Association.

[B69] Rhee Y. S. Yun Y. H. Park S. Shin D. O. Lee K. M. Yoo H. J.. (2008). Depression in family caregivers of cancer patients: the feeling of burden as a predictor of depression. J. Clin. Oncol. 26, 5890–5895. 10.1200/JCO.2007.15.3957 19029423

[B70] Rossi Ferrario S. Zotti A. M. Massara G. Nuvolone G. (2003). A comparative assessment of psychological and psychosocial characteristics of cancer patients and their caregivers. Psychooncology 12, 1–7. 10.1002/pon.626 12548644

[B71] Saba G. W. (2002). L'approccio biopsicosociale: mappe, miti e modelli di salute e malattia, in Il Medico, la Famiglia e la Comunità. L'approccio Biopsicosociale alla Salute e alla Malattia, eds Cigoli V. Mariotti M. (Milano: Franco Angeli), 35–52.

[B72] Saita E. (2009). Psico-Oncologia: Una Prospettiva Relazionale. Milano: Unicopli.

[B73] Saita E. Acquati C. Kayser K. (2015a). Coping with early stage breast cancer: examining the influence of personality traits and interpersonal closeness. Front. Psychol. 6:88. 10.3389/fpsyg.2015.00088 25699003 PMC4318273

[B74] Saita E. Cigoli V. (2009). Le relazioni di coppia e le relazioni familiari nella malattia, in Psico-Oncologia: una Prospettiva Relazionale, ed Saita E. (Milano: Unicopli), 69–88.

[B75] Saita E. De Luca L. Acquati C. (2015b). What is hope for breast cancer patients? A qualitative study. Mediterr. J. Clin. Psychol. III. 10.6092/2282-1619/2015.3.104726585674

[B76] Saita E. Molgora S. Acquati C. (2014). Development and evaluation of the cancer dyads group intervention: preliminary findings. J. Psychosoc. Oncol. 32, 647–664. 10.1080/07347332.2014.955242 25229893

[B77] Saita E. Zanini S. Minetti E. Acquati C. (2016). Best practices to promote patient and donor engagement to care in living donor transplant, in Transformative Healthcare Practice through Patient Engagement, ed Graffigna G. (IGI Global), 1–27.

[B78] Scabini E. Cigoli V. (2012). Alla Ricerca del Famigliare. Il Modello Relazionale-Simbolico. Milano: Raffaello Cortina.

[B79] Scott J. L. Kayser K. (2009). A review of couple-based interventions for enhancing women's sexual adjustment and body image after cancer. Cancer J. 15, 48–56. 10.1097/PPO.0b013e31819585df 19197174

[B80] Stetz K. M. Brown M.-A. (2004). Physical and psychosocial health in family caregiving: a comparison of AIDS and cancer caregivers. Public Health Nurs. 21, 533–540. 10.1111/j.0737-1209.2004.21605.x 15566558

[B81] Traa M. J. De Vries J. Bodenmann G. Den Oudsten B. L. (2014). Dyadic coping and relationship functioning in couples coping with cancer: a systematic review. Br. J. Health Psychol. 20, 85–114. 10.1111/bjhp.12094 24628822

[B82] Vellone E. Chung M. L. Cocchieri A. Rocco G. Alvaro R. Riegel B. (2014). Effects of self-care on quality of life in adults with heart failure and their spousal caregivers: testing dyadic dynamics using the actor-partner interdependence model. J. Fam. Nurs. 20, 120–141. 10.1177/1074840713510205 24189325

[B83] Watson M. Law M. Santos M. Greer S. Baruch J. Bliss J. (1994). The Mini-MAC: further development of the mental adjustment to cancer scale. J. Psychosoc. Oncol. 12, 33–45. 10.1300/J077V12N03_03

